# State Health Officer’s Report*We make a few abstracts from the State Health Officer’s report, just issued, as of interest to the profession at large.

**Published:** 1894-12

**Authors:** R. M. Swearingen

**Affiliations:** State Health Officer; Austin, Texas


### Abstracts and Selections.

### State Health Officer's Report.*

AUSTIN, TEXAS, NOV. 20, 1894.

*To His Excellency, James S. Hogg, Governor of Texas:*

SIR:—I respectfully submit the following report of State quarantine for the years 1893 and 1894:

Your Excellency's proclamations, issued April 2, 1893, and April 2, 1894, respectively, to take effect May 1 of each year, for the protection of the State against the introduction of yellow fever, have been successfully enforced.

The following officers, appointed and commissioned by your Excellency, were assigned to duty at their respective stations on the 1st of May of each year, and with the exception of Dr. Bristow, at Aransas Pass, Dr. Duncan, at Pass Cavallo, and Dr. Weisiger, at Velasco, who were relieved from duty October 1 each year, remained on duty until the close of the quarantine season, November 1. Dr. Weisiger, at Velasco, after having been relieved from duty as quarantine officer, October 1, was assigned to duty, at that point, as quarantine inspector, for the purpose of inspecting trans-Atlantic vessels.

The frequent arrival of vessels at Galveston from cholera-infected countries, made it necessary to retain the quarantine officer at Galveston on duty throughout the year.

QUARANTINE OFFICERS—FOR COAST QUARANTINE.

Dr. A. N. Perkins, Sabine Pass, Jefferson county, assigned to Sabine Pass Station.

Dr. W. F. Blunt, Galveston, Galveston county, assigned to Galveston Station.

D. E. S. Weisiger, Quintana, Brazoria county, assigned to Quintana Station.

Dr. T. G. Duncan, Victoria, Victoria county, assigned to Pass Cavallo Station.

Dr. B. W. Bristow, Flatonia, Fayette county, assigned to Aransas Pass Station.

Dr. A. S. Wolff, Brownsville, Cameron county, assigned to Brazos de Santiago Station.

---

* We make a few abstracts from the State Health Officer's report, just issued, as of interest to the profession at large.

It gives me pleasure to bear testimony to the courage, intelligence and fidelity with which our officers have discharged their duty; at some times arduous and perilous. This is particularly the case at Galveston, where the duties of quarantine officers are most exacting, requiring a practical knowledge of quarantine, a familiarity with epidemic diseases, and the improved methods of preventing them.

Your Excellency also appointed and commissioned a corps of temporary quarantine officers, or inspectors, for the inspection of interstate travel, in accordance with the Quarantine Act of February, 1891, and assigned to duty as follows:

FOR TEMPORARY QUARANTINE.

Dr. W. M. Yandell, El Paso, El Paso county, assigned to El Paso Station.

Dr. M. K. Lott, Eagle Pass, Maverick county, assigned to Eagle Pass Station.

Dr. T. J. Turpin, Laredo, Webb county, assigned to Laredo Station.

On account of the perennial presence of small-pox in Mexico, and the consequent exposure of the western frontier to the contagion, these officers inspect all incoming trains.

SMALL-POX.

The State is at present, and for some months past has been, entirely free from small-pox. This is exceptionally fortunate, because of the great and constant danger of infection to which allusion has just been made. In Mexico, amongst the rural population, the disease is endemic, and there is such an extensive border line between us which it is impossible to guard at all points. Our inspectors, stationed at the three most important thoroughfares—El Paso, Laredo and Eagle Pass—have been vigilant and efficient in their duty; and although the disease has, in several instances, been surreptitiously introduced across the border, it has, in every instance, been promptly extinguished. At Texarkana, however, the disease made its appearance in May, 1894, introduced from Arkansas; and notwithstanding measures were promptly taken for its suppression, the infection spread, and a small epidemic resulted. Pest houses were established on both sides of the State line, one in Arkansas and one in Texas, the latter in charge of Dr. J. W. Talbot, one of your Excellency's commissioned quarantine inspectors. A cordial co-operation between the health officers of both States was established and maintained. The following is a summary of Inspector Talbot's report of the disease:

Total number of cases treated at both county and city small-pox stations, Bowie county and city of Texarkana, all negroes.................................. 7
Total number of deaths.............................. 2
Ratio of deaths........................................ 28.44

At Cleburne, Johnson county, also, the disease made its appearance, the first case having developed before the authorities were aware of its presence. This party communicated the infection to a number of persons, all of whom, when discovered, were isolated and watched, and in all there occurred twenty-three cases. The following is a summary of the report made to this office by Dr. T. C. Osborn, the county physician of Johnson county. In the management of this epidemic Dr. Osborn had the assistance of city physician Dr. J. J. Williamson:

Total number of cases of small-pox at Cleburne .......... 23
Whites, 12; colored, 11; males, 14; females, 9.
Total number of deaths.................................. 5

First case occurred February 23, 1894; last case, April 10th, 1894.

A few sporadic cases of small-pox occurred at intervals at several places elsewhere in the State, but they were promptly isolated, and the infection was quickly suppressed.

In this connection, I beg to refer to a feature in dealing with small-pox, introduced by a conference of State boards of health, respecting interstate notification of the occurrence of certain communicable diseases.

Under the provisions of this agreement, the secretary of every State board of health is notified of the occurrence of small-pox in any State, and its origin, the precautions taken, etc. This notification is without value to us, except as concerning small-pox amongst immigrants. This department has been promptly notified by the health commissioner of New York of the departure for Texas of any immigrants from a small-pox infected or suspected vessel, and in several instances this notification has enabled our local health officer, to whom the information has been transmitted from this office, to identify the party or parties upon arrival, and to keep them under observation until the danger period had been passed. In several instances the introduction of small-pox into Texas has, by this foreknowledge, been prevented.

CHOLERA.

For the past two years much apprehension has been felt lest the Eastern scourge which is now, and for many months past has been, prevailing in parts of Europe and Asia, should be imported into the United States. The system of inspections at the ports of departure, and the sanitary precautions taken with vessels and emigrants before being permitted to sail, inaugurated by the Marine Hospital Service, has reduced the danger to the minimum; and although cholera-infected ships have arrived at New York, the disease has never effected a lodgment on American shores. The danger to Texas was remote, and to be apprehended from interstate communication, as well as by sea. To guard against the former, a medical inspector was appointed by your Excellency for each of the gateways of interstate travel, and assigned to duty. During the season of 1892 and 1893, as long as there was even a remote danger of infection from New Yoek, these officers were kept on duty, and inspected all incoming trains; and although they have not been on active duty this year, they have their camp equipage and outfits stored conveniently, and are prepared at a moment's notice from this office to resume inspections, and, if necessary, establish quarantine.

The following officers, appointed and commissioned as quarantine inspectors, are in readiness at their respective stations to go on duty at short notice, should interstate inspection become necessary at any time:

Dr. Fred C. Combs, Brownsville, Cameron county, assigned to Brownsville Station.

Dr. C. C. Walker, Gainesville, Cooke county, assigned to Gainesville Station.

Dr. W. B. Markham, Denton, Grayson county, assigned to Denison Station.

Dr. J. W. Talbot, Texarkana, Bowie county, assigned to Texarkana Station.

Dr. W. H. Carlisle, Roxton, Lamar county, assigned to Archer Station.

In addition the above Mr. A. L. Webb, not commissioned, was employed temporarily as inspector at Waskom Station.

DIPHTHERIA AND SCARLET FEVER.

A few cases of diphtheria and of scarlet fever were reported to this office as occurring at several points at various times, but by prompt action in isolating them as soon as discovered, and the proper use of disinfectants and other necessary precautions, the disease in no instance became seriously epidemic. At most, only a few cases have occurred in any locality, and as a rule they have been confined to the houses where they originated.

The State Health Officer has, in many instances, at the suggestion or request of the attending physician, visited the interior to confirm or make the diagnosis in any suspected case of infectious disease, and always, upon the arrival of a yellow fever infected ship at any quarantine station, the State Health Officer, having been notified by wire, has made personal inspection of vessel and crew.

CONSUMPTION.

Though not strictly a quarantinable disease, consumption is now classed as a disease "dangerous to public health," and is generally acknowledged to be contagious. Like other diseases of that class, it is preventable, and there is at present much activity in sanitary circles looking to devising means of limiting its spread and consequently diminishing its mortality. Its control is easily within the power and scope of sanitary science, yet public sentiment would revolt at any arbitrary or radical measures if attempted to be enforced for its suppression. It has been thought by leading sanitarians that much can be accomplished in the direction sought, by educating the people, to some extent, in the hygiene of the disease, and the general principles of sanitation, and by pointing out to them the means, very simple within themselves, whereby the danger of contracting the disease, even from one of the same family, may be greatly lessened, if not entirely obviated.

With this object in view, and to co-operate with the health authorities in other States now active in a sanitary campaign against this fatal disease, which, statistics show, is responsible for one-seventh of the total number of deaths, the following circular letter was issued from this office and sent very generally to medical men throughout the State. It was published also in the Texas medical and sanitary journals.

[Circular omitted, as it has already appeared in the JOURNAL. —ED.]

DENGUE.

This disease, which usually occurs in the summer and early fall, and which prevailed as an epidemic in Texas in 1886, has not made its appearance in the State since that year. It would not be mentioned here but for the fact that an outbreak at Key

West in August, 1893 and 1894, was thought by your State Health Officer to be sufficient reason for temporarily interdicting travel and commerce with Key West, which action led to some controversy with the health officials of Florida, and correspondence with the honorable Supervising Surgeon-General of the Marine Hospital Service. Dengue, while not generally regarded as a quarantinable disease, has so often been associated with yellow fever, making its appearance sometimes in Southern cities just prior to that of yellow fever, and so closely resembling that disease in certain manifestations, that its appearance in Key West, concurrently with yellow fever in Havana, with which city Key West is in close proximity, and ordinarily, in close relations, that in the interest of the public health of Texas, it was thought best to take no risk that could be avoided, until sufficient time had elapsed to demonstrate the safety of resuming intercourse. This embargo entailed but slight inconvenience to travel and commerce, and was promptly removed as soon as it was deemed entirely safe to do so.

### THE FRUIT TRADE.

It was thought possible to permit the importation of fruits from the tropics during the quarantine season under proper restrictions, and at the solicitation of certain dealers in New Orleans and Galveston, the experiment was tried. A conference of Gulf State Health Officers was held in New Orleans February 2, 1894, at which were present representatives from all the Southern States. In furtherance of the plan the following was adopted as the conditions upon which a permit should be granted any vessel to engage in the traffic:

[Rules for the regulation of the fruit trade omitted, having previously appeared in the JOURNAL.—ED.]

The Texas Star Mills, of Galveston, was granted a permit under this resolution. They fitted out a steamship, the Giller, for this trade, and she made two voyages to Cuba and certain South American ports. Dr. T. J. McFarland, an experienced yellow fever physician, was commissioned by your Excellency as surgeon of this ship, and accompanied her upon the two voyages, his salary and expenses being paid by the owners of the vessel. Dr. McFarland, on his second voyage, found so much opposition on the part of the officers of the vessel to a rigid enforcement of his instructions, that upon his recommendation the permit to the commander of the Giller was withdrawn, and for that season at least, the enterprise was abandoned.

THE IMPORTATION OF SUGAR AND COFFEE FROM INTERDICTED PORTS.

As you are aware, the importation from Cuba of crude sugar in sacks is carried on to some extent by certain refineries in Texas. With a desire to not interrupt this industry if possible, an effort was made to so load the vessel as to obviate the necessity of detention at quarantine, unloading by lighters, and disinfecting at the quarantine warehouse at Galveston, an undertaking involving an immense amount of labor and expense. Accordingly, special instructions were given for loading vessels at ports of departure, so as to enable us to properly disinfect without shifting cargo. But ship officers, with few exceptions, have thus far failed to sufficiently comply with our requirements, and the old rules, in the majority of arrivals, are still enforced.

THE IMPORTATION OF TURTLES

from intertropical ports was carried on to a small extent at Corpus Christi during a part of the quarantine season, under the following stipulations:

QUARANTINE DEPARTMENT OF TEXAS,
AUSTIN, TEXAS, June 4, 1894.

*Dr. B. W. Bristow, State Quarrntine Officer, Rockport, Texas:*

DEAR DOCTOR:—The turtle-ship must undergo inspection by you before departure for her cargo of turtles; and you must keep on record the names of all persons on board. On return of the ship, require an oath from the officer in command, stating what ports or places have been touched, and see that the same men, and no others, are on board. You can then, if all are well, and they have not been to any interdicted port, allow the transfer of the turtles to the pen which has been prepared for them. The pens should be sufficiently near to permit you to supervise the transfer of the cargo. The men will not be permitted to go on shore, only as may be necessary in the shipment of turtles, nor must any person be permitted to go on board except the pilot, if a pilot be needed. If you know the officer, or can vouch for the correctness of his statements, it will not be necessary to hold the vessel after the discharge of the cargo, if she wishes to return for another shipment of turtles. If they do not wish to return, hold the men in quarantine five days, and then let them go at liberty.

Very respectfully yours,
R. M. SWEARINGEN,
State Health Officer.

THE STATIONS.

A recent inspection of the gulf coast quarantine stations shows them to be in good condition and repair, except that at Pass Cavallo. This station will require some outlay to put it in proper condition for service another season. In this connection I would state that the cost of repairs to stations, and vessels necessarily in use, has been a considerable item in the expense of administering quarantine.

THE QUARANTINE STEAMER HYGIEA.

This vessel, which has been in constant use for four years at Galveston station, has recently been overhauled, cleaned and repaired, at a cost of $558.32. She is now in first class condition for service, and will last, with proper care, many more years.

THE BESSIE ROSS.

The Bessie Ross, a steam vessel purchased for quarantine purposes under a former administration, is lying, and for four years has been, at the wharf at Harrisburg, useless to the service. A watchman is being paid a dollar a day to look after her, and I would recommend that she be sold at auction for what she will bring. The last legislature authorized the sale of the vessel, but up to date I have not been able to dispose of her.

U. S. GOVERNMENT SUPERVISION OF STATE QUARANTINE.

It affords me pleasure to state that our relations with the United States quarantine authorities, administered by the Marine Hospital Service, have been pleasant and satisfactory, and no clash of opinion or authority has occurred. Under orders of the Supervising Surgeon-General, Surgeon J. M. Gassaway, of the Marine Hospital Service, has twice inspected the stations on the coast, and has made a favorable report upon the efficiency of their management.

COUNTY PHYSICIANS.

Section 14 of the Quarantine law, passed in February, 1891, makes it the duty of every county judge within the State of Texas, after each general election of State and county officers, or as soon thereafter as practicable, to select from the physicians of the respective counties, one of high character and recognized ability, who shall be known as the county physician. In the same section are defined the duties of said county physician, and it is stipulated, amongst others, that he "shall, in all quarantines, establish rules in harmony and accord with the rules prescribed by the State Health Officer; shall respect and obey instructions from said officer, and make written reports to him of their official acts whenever required to do so, giving cause and history of epidemic, number of deaths, etc."

I beg to call your Excellency's attention to the fact that very generally this requirement of the county judges is neglected, and county physicians have not been appointed in more than about one-third of the counties of the State, and in some of these only after personal solicitation; in some cases repeated by letter to the judge, from this office. As late as February 20th, 1893, finding that there were so many counties not provided with a county physician, as required by law, I addressed the following circular letter to the judges of these counties. In response to this letter a number of appointments were made; but there are still many counties in which no physician has yet been appointed.

CIRCULAR LETTER TO COUNTY JUDGES.

QUARANTINE DEPARTMENT OF TEXAS,
AUSTIN, TEXAS, February 20, 1893.

To the County Judge of .......... County,
.............. Texas.

DEAR SIR:—Your attention is respectfnlly asked to section 14 of the present quarantine law. It was enacted by the Twenty-second Legislature, and is entitled "An Act to Regulate the Establishment of Quarantine in Texas." If you have not already done so, please comply with its requirements, and at your earliest convenience report to this office the name and address of your appointee. The threatening aspect of the cholera renders it necessary that the Quarantine Department of Texas shall be thoroughly equipped and ready for immediate action in any emergency. You are requested to act with promptness, and I trust you have a proper appreciation of your responsibility in the premises.

Very respectfully yours,
R. M. SWEARINGEN, M. D.,
State Health Officer.

As the station buildings, boats and wharves, belonging to the Quarantine Department, are now in good condition, the outlay for the next two years to keep them in repair, will be comparatively small. I respectfully submit that forty thousand dollars ($40,000) for each year be appropriated to cover all expenses of State quarantine, and that two thousand dollars ($2,000) per annum additional be appropriated (or as much thereof as may be necessary) to employ an expert in microscopy and chemistry, to make analyses of suspected polluted waters, and bacteriological examinations, whenever, in the judgment of the State Health Officer, such examinations are deemed necessary to protect the public health.

The wonderful advances in the science of preventive measures against endemic and epidemic diseases within the last few years imposes this requirement, and without it no health department is thoroughly equipped to perform the responsible duties that devolve upon it.

Deeply grateful to your Excellency for the support given, and the great kindness at all times extended to me,

I have the honor to be, your obedient servant,

R. M. SWEARINGEN,
State Health Officer.

